# The association of self-esteem, depression and body satisfaction with obesity among Turkish adolescents

**DOI:** 10.1186/1471-2458-7-80

**Published:** 2007-05-16

**Authors:** Dilek Ozmen, Erol Ozmen, Dilek Ergin, Aynur Cakmakci Cetinkaya, Nesrin Sen, Pinar Erbay Dundar, E Oryal Taskin

**Affiliations:** 1 Department of Public Health Nursing, School of Health, Celal Bayar University, Manisa, Turkey; 2 Department of Psychiatry, School of Medicine, Celal Bayar University, Manisa, Turkey; 3 Department of Paediatric Nursing, School of Health, Celal Bayar University, Manisa, Turkey; 4 Department of Public Health, School of Medicine, Celal Bayar University, Manisa, Turkey

## Abstract

**Background:**

The purpose of this study was to determine the prevalence of overweight and obesity and to examine the effects of actual weight status, perceived weight status and body satisfaction on self-esteem and depression in a high school population in Turkey.

**Methods:**

A cross-sectional survey of 2101 tenth-grade Turkish adolescents aged 15–18 was conducted. Body mass index (BMI) was calculated using weight and height measures. The overweight and obesity were based on the age- and gender-spesific BMI cut-off points of the International Obesity Task Force values. Self-esteem was measured using the Rosenberg Self-Esteem Scale, and depression was measured using Children's Depression Inventory. Logistic regression analysis was used to examine relationships among the variables.

**Results:**

Based on BMI cut-off points, 9.0% of the students were overweight and 1.1% were obese. Logistic regression analysis indicated that (1) being male and being from a higher socio-economical level were important in the prediction of overweight based on BMI; (2) being female and being from a higher socio-economical level were important in the prediction of perceived overweight; (3) being female was important in the prediction of body dissatisfaction; (4) body dissatisfaction was related to low self-esteem and depression, perceived overweight was related only to low self-esteem but actual overweight was not related to low self-esteem and depression in adolescents.

**Conclusion:**

The results of this study suggest that school-based adolescents in urban Turkey have a lower risk of overweight and obesity than adolescents in developed countries. The findings of this study suggest that psychological well-being of adolescents is more related to body satisfaction than actual and perceived weight status is.

## Background

It is a well-recognised fact that obesity is a major public health problem in the world, and the prevalence of obesity is increasing in both developed and developing countries [1,2]. Although the prevalence of overweight and obesity varies between different countries and ethnic groups, adolescent obesity is also one of the major health problems all over the world [3,4].

Comparison of cross-sectional data from Israel, United States and 13 European countries has shown that the prevalence of overweight, defined as a BMI above the 85^th ^centile and below 95^th ^centile varied between 5.2% and 28.9% for boys and 8.1% and 31.0% for girls; the prevalence of obesity defined as a BMI above 95th centile varied between 1.9% and 13.9% for boys and 1.1% and 15.1% for girls among adolescents [5]. The prevalence of overweight and obesity (excess of the 85^th ^and 95^th ^percentiles) has been found to be 21.1% and 7.8% among Iranian adolescents [6], 19.8% and 7.9% among Mexican adolescents, 12.1% and 6.2% among Egyptian adolescents [7], 15.9% and 18.4% among Bahraini adolescents [8] respectively. The prevalence of overweight ranged from 10.3% to 12.0% and obesity from 1.6% to 3.6% among Turkish adolescents [9-12].

Although obesity is a prevalent disorder, its social determinants and psychosocial consequences have not yet been fully understood, because obesity is a heterogeneous disorder of multiple aetiology [13]. Adolescence is a critical developmental phase characterised by dramatic physical and psychological changes. Physical changes and appearance are important preoccupations of adolescents, and for this reason obesity may predispose them to mental health consequences. But there is no consistent finding in the literature concerning the relationship between obesity, mental health and psychopathology [14,15]. The relationship between self-esteem and obesity was unclear whether self-esteem was consistently related to obesity [15]. In general, clinic-based studies have found a relationship between overweight and depression more often than have population-based studies [8,16]. In addition, it is not fully known if certain variables such as demographic variables, physical appearance, body satisfaction and self image have an effect on overweight or not. Perceived overweight adolescents were more likely to experience anxiety and depression than perceived normal and underweight adolescents [17]. On the other hand, many overweight adolescents are socially marginalized. Dissatisfaction with body and discrimination may aggravate the social and emotional consequences of overweight in this age group [18].

Although adolescent obesity is a prevalent disorder all over the world, there are only a few studies which have examined the prevalence of adolescent overweight and its psychological consequences in Turkey. The purpose of this study was to determine the prevalence of obesity and to examine the effects of actual weight status, perceived weight status and body satisfaction on self-esteem and depression in a high school population in Turkey.

## Methods

### Study population

The subjects of the study were tenth-grade students of all public and private high schools in Manisa, a city located in western part of Turkey. There were a total of 9 schools (6 public and 3 private schools) and 2444 tenth-grade students in the schools according to the schools' records in Manisa. All of the tenth-grade students were sample of this study, but 241 students were absent at the school at the time of applying date of the questionnaire. Therefore the questionnaire was applied to 2203 students. No subject refused to participate in the study. But because of the absence of answers for some questions, the data of eighty-one subjects and because of the adolescent stage mostly ends with the age of 18; the data regarding twenty-one subjects who were older than 18 years old were excluded. Two thousand one hundred and one students finally constituted the sample of this study. The students ranged from 15 to 18 years in age, with a mean age of 16.55 (SD = 0.66). Approval was obtained from the state Department of School Education and consent was obtained from the school principals and students.

### Measures

The BMI was calculated for each student by using the standard formula: the weight in kilograms was divided by the square of height in meters. The international BMI age- and gender-specific cut-off points (based on pooled international data and linked to the widely used adult cut off points of a body mass index of 25 and 30 kg/m^2^) were used to define overweight and obesity [19].

Self esteem was measured by using the Rosenberg Self-Esteem Scale (SES). The SES is a self-report questionnaire and consists of 10 items. Five of the items are phrased as positive and five of them as negative statements. The total score of the scale yields 0 to 6, where 0 represents high and 6 represents low self-esteem [20]. Cronbach's alpha of SES was 0.8024 in the present study.

Depression was measured by using the Children's Depression Inventory (CDI), which is used as a scale for self-report assessment of depression in children and adolescents and consists of 27 likert-type items ranging from 0 to 2. The total score of the scale yields 0 to 54, where higher scores reflect severity of depression and a score of > 19 is the criterion score for identifying clinical depression [21,22]. Cronbach's alpha of the CDI was 0.7827 in the present study.

We asked the students where they had mostly lived (in a rural, suburban, or urban area) up to the applying date of the questionnaire, and recorded their answers as residential background. Perceived weight status was evaluated by one question: "How do you perceive yourself?". Body satisfaction was evaluated by one question: "Are you satisfied with your body?".

Prior to data collection, informed consent was obtained verbally from the students. The questionnaires were administered in the classrooms. Trained nursing school students measured and recorded each student's weight, height, sex, and age.

### Statistical analysis

Data were analysed descriptively to determine the demographic and basic characteristics (BMI weight status, perceived weight status, body satisfaction) of the sample. In addition to the descriptive analyses, logistic regression analysis was performed to find out relationships among the demographic features, BMI weight status, perceived weight status, depression, self-esteem and body satisfaction. Logistic regression analysis was performed at the first step to find out the effects of demographic features on weight status, perceived weight status, and body satisfaction. Demographic features were accepted as independent variables and BMI weight status, perceived weight status, and body satisfaction were accepted as dependent variables; logistic regression analysis was separately performed, one by one, for each of them. Then logistic regression analysis was performed again to find out the effects of weight status based on BMI and of perceived weight status on depression, self esteem and body satisfaction. Body satisfaction, depression and self-esteem were individually accepted as dependent variables. In addition to the remaining variables, gender and socio-economic status were taken as independent variables.

## Results

The socio-demographic features of the sample are given in Table 1. The distributions for BMI weight status, perceived weight status and body satisfaction are presented in Table 2. Based on BMI, 9.0% of the students were overweight and 1.1% were obese, 15.2% of the students perceived themselves as fat and 47.2% of the students were dissatisfied with their body. While 3.1% of non-overweight and 40.7% of overweight boys perceived themselves as fat, 17.3% of non-overweight and 77.8% of overweight girls perceived themselves as fat (Table 3).

**Table 1:** Socio-demographic features of the sample

	n	%
Age		
15	56	2.7
16	973	46.3
17	919	43.7
18	153	7.3

Gender		
Male	1050	50.0
Female	1051	50.0

Perceived socio-economic status		
Lower	160	7.6
Middle	1809	86.1
Upper	132	6.3

Residential background		
Urban	1637	77.9
Suburban	171	8.1
Rural	293	13.9

Mother's educational level		
Uneducated	191	9.1
Primary school graduate	989	47.1
Secondary school graduate	246	11.8
High school graduate	474	22.5
University graduate	201	9.5

Father's educational level		
Uneducated	70	3.3
Primary school graduate	723	34.4
Secondary school graduate	297	14.1
High school graduate	569	27.1
University graduate	442	21.1

Total	2101	100.0

**Table 2:** BMI weight status, perceived weight status and body satisfaction of the Turkish adolescents

	Male		Female		Total	
	n	%	n	%	n	%

BMI weight status						
Non-overweight	930	88.6	958	91.2	1888	89.9
Overweight	108	10.3	81	7.7	189	9.0
Obesity	12	1.1	12	1.1	24	1.1

Perceived weight status						
Thin	174	16.6	133	12.7	307	14.6
Normal weight	795	75.7	679	64.6	1474	70.2
Fat	81	7.7	239	22.7	320	15.2

Body Satisfaction						
Satisfied	655	62.4	455	43.3	1110	52.8
Dissatisfied	395	37.6	596	56.7	991	47.2

**Table 3:** The relationship between perceived and actual weight status

	Perceived weight status		
	
Actual weight status	Thin (%)	Normal weight (%)	Fat (%)
Boys			
Non-overweight	173 (18.6)	728 (78.3)	29 (3.1)
Overweight	1 (0.9)	63 (58.3)	44 (40.7)
Obese	0 (0.0)	4 (33.3)	8 (66.7)

Girls			
Non-overweight	133 (13.9)	659 (68.8)	166 (17.3)
Overweight	0 (0.0)	18 (22.2)	63 (77.8)
Obese	0 (0.0)	2 (16.7)	10 (83.3)

### The effect of demographic features on overweight based on BMI, perceived overweight and body satisfaction

The effect of demographic features on overweight based on BMI, perceived overweight and body satisfaction was analysed by using logistic regression analysis. It was seen that gender and socio-economic level had a significant effect on overweight based on BMI, but residential background, mother's and father's educational level did not. Being male and being from higher socio-economical level predicted overweight based on BMI (Table 4).

**Table 4:** The effect of demographic features on overweight and body dissatisfaction

	p	B	OR	95% CI
Overweight based on BMI				
Residential background	0.128	0.311	1.365	0.914–2.039
Mother's educational level	0.116	-0.289	0.749	0.522–1.074
Father's educational level	0.157	-0.260	0.771	0.538–1.105
Socio-economic status	0.002	0.725	2.064	1.294–3.292
Gender	0.028	0.327	1.387	1.037–1.856

Perceived overweight				
Residential background	0.629	-0.080	0.923	0.667–1.277
Mother's educational level	0.191	0.209	1.232	0.901–1.683
Father's educational level	0.438	0.119	1.126	0.834–1.521
Socio-economic status	0.003	-0.655	0.520	0.337–0.802
Gender	0.000	1.280	3.597	2.737–4.728

Body dissatisfaction				
Residential background	0.385	0.097	1.102	0.885–1.370
Mother's educational level	0.646	-0.054	0.948	0.753–1.192
Father's educational level	0.768	-0.032	0.968	0.781–1.200
Socio-economic status	0.397	0.160	1.173	0.811–1.698
Gender	0.000	0.787	2.196	1.841–2.619

In logistic regression analysis, it was seen that gender and socio-economic level had a significant effect on perceived overweight, but residential background, mother's and father's educational levels did not. Being female and being from higher socio-economical levels predicted perceived overweight (Table 4).

In logistic regression analysis it was seen that only gender had a significant effect on body satisfaction; residential background, socio-economical level, mother's and father's educational level did not. The female students were more dissatisfied with their body (Table 4).

### The effects of BMI on depression, self-esteem and body satisfaction

In logistic regression analysis it was seen that overweight based on BMI did not have a significant effect on depression and self-esteem, but had on body satisfaction. Overweight adolescents were more dissatisfied with their body (Table 5).

**Table 5:** The effect of overweight based on BMI on depression, self-esteem and body satisfaction

	p	B	OR	95% CI
Depression				
Gender	0.063	0.328	1.388	0.983 – 1.960
Socio-economic status	0.842	0.072	1.075	0.526 – 2.198
Overweight based on BMI	0.075	0.561	1.744	0.955 – 3.230
Perceived weight status	0.183	0.078	1.361	0.865 – 2.141
Satisfaction with body	0.000	0.688	1.990	1.379 – 2.871
Self Esteem	0.000	-2.309	0.100	0.060 – 0.160

Self-esteem				
Gender	0.100	0.445	1.560	0.918 – 2.652
Socio-economic status	0.259	-0.840	0.432	0.101 – 1.853
Overweight based on BMI	0.708	-0.152	0.859	0.387 – 1.906
Perceived weight status	0.008	-0.860	0.423	0.224 – 0.800
Satisfaction with body	0.013	-0.776	0.460	0.250 – 0.847
Depression	0.000	-2.300	0.100	0.060 – 0.167

Body dissatisfaction				
Gender	0.000	0.551	1.735	1.431 – 2.103
Socio-economic status	0.011	0.564	1.759	1.140 – 2.712
Overweight based on BMI	0.010	-0.528	0.590	0.396 – 0.879
Perceived weight status	0.000	2.810	16.617	10.338 – 26.711
Depression	0.000	0.671	1.956	1.350 – 2.833
Self Esteem	0.018	-0.742	0.476	0.257 – 0.882

### The effects of perceived weight status on depression, self-esteem and body satisfaction

Perceived weight status had significant effects on self-esteem and body satisfaction, but not on depression. The students who perceived themselves as fat had lower self esteem and were dissatisfied with their bodies (Table 5).

### The effects of body satisfaction on depression and self-esteem

Body satisfaction had significant effects on self-esteem and depression. The students who were dissatisfied with their bodies had low self-esteem and were depressive (Table 5).

## Discussion

In this study, it was found that (i) the prevalence of overweight and obesity is lower in Turkish school-based adolescents than in adolescents in developed countries, (ii) overweight based on BMI was associated with gender and socio-economical level (iii) there was a clear discrepancy between adolescents' perceived weight and actual weight, (iv) overweight based on BMI did not have a significant effect on depression and self-esteem, but had on body satisfaction, (v) perceived weight status had significant effects on self esteem and body satisfaction, but not on depression, and (vi) body satisfaction predicted self-esteem and depression better than weight status based on BMI and perceived weight status.

In this study it was seen that the prevalence of overweight and obesity based on BMI was 10.3% and 1.1% respectively for adolescent boys, and 7.7% and 1.1% for adolescent girls. In the other studies conducted in Turkey, the prevalence of overweight ranged from 10.3% to 12.0% and obesity 1.6%–3.6% among Turkish adolescents [9-12]. But the prevalence of overweight and obesity defined by using IOTF cut off points [19] was found to be higher in developed countries and in some other developing countries. The prevalence of overweight was 22.7% (17.5% of moderate overweight and 5.25% of obesity) in sixth-grade French adolescents [23]; and another study showed that 15% of 11–16-year-old Canadian youth were overweight and 4.6% were obese [24]. In Great Britain, the prevalence of obesity and overweight was 4.0% and 15.4% respectively [25]. In a low-income Mexican American population, 40.1% (22.1% were obese, 18% were at risk for obesity) of adolescents aged between 12 and 17 had a BMI at the 85^th ^percentile or higher [26]. Booth *et al. *analysed three independent surveys in Australia, and found that some 19–23% of Australian children and adolescents were either overweight or obese [27]. In Taiwan, the prevalence of overweight and obesity was respectively 17.6% and 3.7% in boys and 9.4% and 1.6% in girls [28]. In Greek school-aged children and adolescents, 9.1% of the girls and 21.7% of the boys were classified as overweight and 1.2% of the girls and 18.8% of the boys were classified as obese [29].

In Sweden, it was found that 11.6% and 11.4% of the boys and 5.5% and 4.8% of the girls aged between 15 and 18 were overweight based on BMI value ≥91^st ^percentile and 8.9% and 7.3% of the boys and 4.2% and 3.9% of the girls were obese based on BMI value ≥98^th ^percentile [30]. The overall prevalence of overweight and obesity (excess of the 85^th ^and 95^th ^percentiles) was found to be 21.1% and 7.8% respectively in adolescent Tehrani students [6]. Among the Mexican and Egyptian adolescents, the prevalence of overweight (excess of the 85^th ^percentile) was 19.8% and 12.1% and the prevalence of obesity (excess of the 95^th ^percentile) was 7.9% and 6.2% respectively [7].

These results suggest that adolescents in urban Turkey have lower risk of overweight and obesity than adolescents in developed countries and in some other developing countries. Turkish adolescents may have different dietary and exercise habits. But the prevalence of overweight was still high and must be taken into account. The lifestyle of the adolescents in urban Turkey has changed during recent years: television viewing, computer use and intake of fast food and this cultural change may increase the prevalence of overweight in the future.

It was seen that being male and being from higher socio-economical level were predictors of overweight based on BMI in this study. Similarly the prevalence of overweight and obesity was found to be higher in boys than in girls in Swedish [30], Canadian [24], Taiwanese [28] and Greek [29] adolescents. Unlike these results, in the studies conducted in adolescent Tehrani and Egyptian students, the prevalence of overweight among girl students was found higher than among boys [6,7]. Similarly in a study conducted in a representative sample of schools in the United States, it was found that adolescents from more advantaged family backgrounds (with at least one college-educated parent and a higher family income) were less likely to be overweight [31]. In the Mexican and Egyptian adolescents, a higher prevalence of overweight and obesity was found in the highest socioeconomic status in both sexes [7]. In contrast to our findings, it was found that overweight was more frequent in low economic zones in sixth-grade French adolescents [23]. In an Australian study, it was found that adolescents of lower socioeconomic status were more likely to be overweight [32]. These results suggest that the effects of social factors may be complex and aspects of these effects may vary with cultural features. On the other hand, these findings suggest that thinness is more important for girls than boys in all cultures, and that adolescent boys have a higher risk of being overweight than adolescent girls.

Consistent with other studies [8,17,33], in this study it was determined that there was a discrepancy between adolescents' perceived weight status and their actual weight. Girls had a tendency to perceive themselves as fat more than did boys, and boys had a tendency to underestimate their overweight. Similar tendencies have been seen among Chinese, Bahraini and Korean adolescents [8,17,33]. These results suggest that thinness is related to beauty and women want to be thin more than men, all over the world. A high prevalence of overweight in boys may be due to underestimation of body weight.

The findings about the relationship between overweight and psychopathology are inconsistent in the literature. Meta-analytic studies suggest no significant relationship between obesity and depression in the population [34]. In our study it was determined that overweight based on BMI had no effect on self-esteem and depression. Kim & Kim similarly stated that weight status based on BMI did not help predict the level of self-esteem and depression [35]. Wardle et al. also stated that regardless of gender, socioeconomic status or ethnicity, reports of depressive symptoms were not significantly higher in obese than in normal-weight groups [36]. Renman *et al. *have found that obesity had only a minor correlation with self-esteem [37]. The self-esteem and anxiety of obese students did not differ from those of normal weight ones [16]. But in some clinical populations, a higher ratio of psychopathology (depression, behavioural problems, low self-esteem) was reported [38]. French *et al. *reviewed the literature to find out the relationship between self-esteem and obesity, and found that it was unclear whether self-esteem was consistently related to obesity [15]. There may be multiple patterns of association between obesity-psychopathology or a subgroup of obese adolescents may be an at-risk population. Needham & Crosnoe found that overweight status was related to depressive symptoms among girls and younger adolescents of both genders [31]. On the other hand, as mentioned by Fabricatore & Wadden, obesity may not be systematically associated with psychopathological conditions [39]. There may be some other factors which can mediate the relationship between obesity and psychological distress. Friedman *et al. *reported that body-image satisfaction partially mediated the relationship between degree of overweight and depression/self-esteem [40]. Pesa *et al. *also stated that body image might explain the low self-esteem of overweight female adolescents [41]. In the present study, body dissatisfaction was associated with low self-esteem and depression, and perceived overweight was associated with low self-esteem, but not with depression. On the other hand, actual weight was not associated with low self-esteem and depression. These findings suggest that psychological well-being is more related with body satisfaction than actual and perceived weight status.

## Conclusion

The results of this study have implications for school health in Turkey. Adolescents need more knowledge to distinguish between healthy and unrealistic weight standards. But to focus only to actual and perceived weight status is insufficient to prevent depression and lowered self esteem. School health programs towards obesity must be different for girls and for boys. In boys, the program should primarily address their underestimation of overweight. But in girls, as it was mentioned by Pesa et al [41], efforts should be directed toward encouraging and supporting a recognition of personal strengths not related to physique.

The results of this study suggest that the prevalence of overweight and obesity is lower in Turkish school-based adolescents than in developed countries, but prevalence is still high, which must be taken into account. Although people have traditionally valued moderate fatness in Turkey, the results of this study suggest that traditional values are changing, especially for girls. The lifestyle of adolescents in urban Turkey is also changing and coming to resemble that of developed countries which has been considered to be responsible for obesity. Therefore, preventive actions must be planned and initiated in Turkey from now on.

## Competing interests

The author(s) declare that they have no competing interests.

## Authors' contributions

DO conceived of the study, performed the statistical analysis, and drafted the manuscript. EO participated in the design of the study, and helped to draft the manuscript. DE participated in the design of the study, data collection, revision of the paper. ACC participated in design of the study, data collection, statistical analysis. NS participated in design of the study, data collection. PED supervised the statistic analysis and interpretation of results. EOT participated in design of the study and helped to draft the manuscript. All authors have read and approved the final manuscript.

## Pre-publication history

The pre-publication history for this paper can be accessed here:



## References

[B1] Reilly JJ (2006). Obesity in childhood and adolescence: evidence based clinical and public health perspectives. Postgrad Med J.

[B2] Chinn S, Rona RJ (2001). Prevalence and trends in overweight and obesity in three cross sectional studies of British Children, 1974–94. BMJ.

[B3] Musaiger AO (2004). Overweight and obesity in the Eastern Mediterranean Region: can we control it?. East Mediterr Health J.

[B4] Speiser PW, Rudolf MCJ, Anhalt H, Camacho-Hubner C, Chiarelli F, Eliakim A, Freemark M, Gruters A, Hershkovitz E, Iughetti L, Krude H, Latzer Y, Lustig RH, Pescovitz OH, Pinhas-Hamiel O, Rogol AD, Shalitin S, Sultan C, Stein D, Vardi P, Werther GA, Zadik Z, Zuckerman-Levin N, Hochberg Z, Obesity Consensus Working Group (2005). Childhood obesity. J Clin Endocrinol Metab.

[B5] Lissau I, Overpeck MD, Ruan WJ, Due P, Holstein BE, Hediger ML (2004). Body mass index and overweight in adolescents in 13 European countries, Israle, and United States. Arch Pediatr Adolesc Med.

[B6] Mohammadpour-Ahranjani B, Rashidi A, Karandish M, Eshraghian MR, Kalantari N (2004). Prevalence of overweight and obesity in adolescent Tehrani students, 2000–2001: an epidemic health problem. Public Health Nutr.

[B7] Salazar-Martinez E, Allen B, Fernandez-Ortega C, Torres-Mejia G, Galal O, Lazcano-Ponce E (2006). Overweight and obesity status among adolescents from Mexico and Egypt. Arch Med Res.

[B8] Al-Sendi AM, Shetty P, Musaiger AO (2004). Body weight perception among Bahraini adolescents. Child Care Health Dev.

[B9] Oner N, Vatansever U, Sari A, Ekuklu G, Guzel A, Karasalihoglu S, Boris NW (2004). Prevalence of underweight, overweight and obesity in Turkish adolescents. Swiss Med Wkly.

[B10] Uckun-Kitapci A, Tezic T, Firat S, Sipahi T, Barrier R, Edwards LJ, Calikoglu AS (2004). Obesity and type 2 diabetes mellitus: a population-based study of adolescents. J Pediatr Endocrinol Metab.

[B11] Sur H, Kolotourou M, Dimitriou M, Kocaoglu B, Keskin Y, Hayran O, Manios Y (2005). Biochemical and behavioral indices related to BMI in schoolchildren in urban Turkey. Prev Med.

[B12] Krassas GE, Tsametis C, Baleki V, Constantinidis T, Unluhizarci K, Kurtoglu S, Kelestimur F, Balkan Group for the Study of Obesity (2004). Prevalence of overweight and obesity among children and adolescents in Thessaloniki-Greece and Kayseri-Turkey. Pediatr Endocrinol Rev.

[B13] Wadden TA, Kaplan HI, Sadock BJ (1995). Obesity. Comprehensive Textbook of Psychiatry/VI.

[B14] Wadden TA, Stunkard AJ (1985). Social and psychological consequences of obesity. Ann Intern Med.

[B15] French SA, Story M, Perry CL (1995). Self-esteem and obesity in children and adolescents: a literature review. Obes Res.

[B16] Pastore DR, Fisher M, Friedman SB (1996). Abnormalities in weight status, eating attitudes, and eating behaviours among urban high school students correlation with self-esteem and anxiety. J Adolesc Health.

[B17] Xie B, Liu C, Chou CP, Xia J, Spruijt-Metz D, Gong J (2003). Weight perception and psychological factors in Chinese adolescents. J Adolesc Health.

[B18] Strauss RS, Pollack HA (2003). Social marginalization of overweight children. Arch Pediatr Adolesc Med.

[B19] Cole TJ, Bellizzi MC, Flegal KM, Dietz WH (2000). Establishing a standard definition for child overweight and obesity worldwide: international survey. BMJ.

[B20] Oner N (1997). The Psychological Tests Which Are Used In Turkey.

[B21] Kovacs M (1985). The Chidren's Depression Inventory (CDI). Psychopharmacol Bull.

[B22] Oy B (1990). Depression rating scale for children: a validity and reliability study. Turk Psikiyatri Derg.

[B23] Klein-Platat C, Wagner A, Haan MC, Arveiler D, Schlienger JL, Simon C (2003). Prevalence and sociodemographic determinants of overweight in young French adolescents. Diabetes Metab Res Rev.

[B24] Janssen I, Katzmarzyk PT, Boyce WF, King MA, Pickett W (2004). Overweight and obesity in Canadian adolescents and their associations with dietary habits and physical activity patterns. J Adolesc Health.

[B25] Jebb SA, Rennie KL, Cole TJ (2004). Prevalence of overweight and obesity among young people in Great Britain. Public Health Nutr.

[B26] Lacar ES, Soto X, Riley WJ (2000). Adolescent obesity in a low-income Mexican American District in South Texas. Arch Pediatr Adolesc Med.

[B27] Booth ML, Wake M, Armstrong T, Chey T, Hesketh K, Mathur S (2001). The epidemiology of overweight and obesity among Australian children and adolescents, 1995–97. Aust N Z J Public Health.

[B28] Page RM, Lee CM, Miao NF (2004). Assessing prevalence of overweight and obesity through self-reports of height and weight by high school students in Taipe, Taiwan. J Sch Health.

[B29] Karayiannis D, Yannakoulia M, Terzidou M, Sidossis LS, Kokkevi A (2003). Prevalence of overweight and obesity in school-aged children and adolescents. Eur J Clin Nutr.

[B30] Berg IM, Simonsson B, Brantefors B, Ringqvist I (2001). Prevalence of overweight and obesity in children and adolescents in a county in Sweden. Acta Paediatr.

[B31] Needham BL, Crosnoe RC (2005). Overweight status and depressive symptoms during adolescence. J Adolesc Health.

[B32] O'Dea JA, Caputi P (2001). Association between socioeconomic status, weight, age and gender, and body image and weight control practices of 6- to 19-year-old children and adolescents. Health Educ Res.

[B33] Kim O, Kim K (2003). Comparisons of body mass index, perception of body weight, body shape satisfaction, and self-esteem among Korean adolescents. Percept Mot Skills.

[B34] Faith MS, Matz PE, Jorge MA (2002). Obesity-depression associations in the population. J Psychosom Res.

[B35] Kim O, Kim K (2001). Body weight, self-esteem, and depression in Korean Female Adolescents. Adolescence.

[B36] Wardle J, Williamson S, Johnson F, Edwards C (2006). Depression in adolescent obesity: cultural moderators of the association between obesity and depressive symptoms. Int J Obes (Lond).

[B37] Renman C, Engström I, Silfverdal SA, Aman J (1999). Mental health and psychosocial characteristics in adolescents in adolescent obesity: a population-based case control study. Acta Paediatr.

[B38] Erermis S, Cetin N, Tamar M, Bukusoglu N, Akdeniz F, Goksen D (2004). Is obesity a risk factor for psychopathology among adolescents?. Pediatr Int.

[B39] Fabricatore AN, Wadden TA (2004). Psychological aspects of obesity. Clin Dermatol.

[B40] Friedman KE, Reichmann SK, Costanzo PR, Musante GJ (2002). Body image partially mediates the relationship between obesity and psychological distress. Obes Res.

[B41] Pesa JA, Syre TR, Jones E (2000). Psychosocial differences associated with body weight among female adolescents: the importance of body image. J Adolesc Health.

